# Measurements to predict the time of target replacement of a helical tomotherapy

**DOI:** 10.1120/jacmp.v12i4.3596

**Published:** 2011-11-15

**Authors:** Severin Kampfer, Stefan Schell, Marciana N. Duma, Jan J. Wilkens, Peter Kneschaurek

**Affiliations:** ^1^ Department of Radiation Oncology Technische Universität München, Klinikum rechts der Isar München Germany

**Keywords:** helical tomotherapy, treatment beam, target replacement, depth‐dose curve, energy spectrum

## Abstract

Intensity‐modulated radiation therapy (IMRT) requires more beam‐on time than normal open field treatment. Consequently, the machines wear out and need more spare parts. A helical tomotherapy treatment unit needs a periodical tungsten target replacement, which is a time consuming event. To be able to predict the next replacement would be quite valuable. We observed unexpected variations towards the end of the target lifetime in the performed pretreatment measurements for patient plan verification. Thus, we retrospectively analyze the measurements of our quality assurance program. The time dependence of the quotient of two simultaneous dose measurements at different depths within a phantom for a fixed open field irradiation is evaluated. We also assess the time‐dependent changes of an IMRT plan measurement and of a relative depth dose curve measurement. Additionally, we performed a Monte Carlo simulation with Geant4 to understand the physical reasons for the measured values. Our measurements show that the dose at a specified depth compared to the dose in shallower regions of the phantom declines towards the end of the target lifetime. This reproducible effect can be due to the lowering of the mean energy of the X‐ray spectrum. These results are supported by the measurements of the IMRT plan, as well as the study of the relative depth dose curve. Furthermore, the simulation is consistent with these findings since it provides a possible explanation for the reduction of the mean energy for thinner targets. It could be due to the lowering of low energy photon self‐absorption in a worn out and therefore thinner target. We state a threshold value for our measurement at which a target replacement should be initiated. Measurements to observe a change in the energy are good predictors of the need for a target replacement. However, since all results support the softening of the spectrum hypothesis, all depth‐dependent setups are viable for analyzing the deterioration of the tungsten target. The suggested measurements and criteria to replace the target can be very helpful for every user of a TomoTherapy machine.

PACS numbers: 87.55.N‐, 87.55.Qr, 87.55.T‐, 87.56.bd

## I. INTRODUCTION

Recent advancements in radiotherapy were driven by new treatment methods based on intensity‐modulated radiation therapy (IMRT). These techniques usually need more beam‐on time than open field treatments, which puts a higher burden onto the acceleration machine. It is widely known that the tungsten target of the TomoTherapy Hi·Art system (TomoTherapy, Madison, WI, USA,^(^ ^1^ ^)^) suffers from wear effects^(^ ^2^ ^–^ ^5^ ^)^ and needs periodic replacement. This replacement normally takes a couple of days, including the time for delivering the spare parts. In our clinic, the target needs replacement on average every 10 months and the whole process usually takes three days. Since so far a target breakdown is an unforeseen event, it results in additional work for the staff and longer overall treatment times for the patients. The clinical impact of an unplanned break within the therapy schedule should also be taken into account. Staton et al.^(^ ^5^ ^)^ found possible dose deviations between the planned and the delivered treatment of up to 4.5% shortly before the target replacement took place.

The question we want to address is: can the breakdown of the target be predicted? If so, the downtime of the machine could be reduced to the time of performing the mechanical work and dosimetry and could be planned beforehand. In this study, we retrospectively analyze measurements and present a method to predict the point in time of future target replacements. Additionally, we present a correlation of measurements to the wear out observed in broken targets.

## II. MATERIALS AND METHODS

We analyzed three target replacements in our clinic. Before each breakdown, unusual but reproducible results of our standard QA measurements were noted. Furthermore, we observed that the worn out targets were thinner than the new ones. From various types of checks that we routinely carry out in the clinic, we present three checks that analyze the long‐term stability of the system. In order to understand the physics behind the measured effects, we additionally performed a Monte Carlo simulation.

### A. Relative dose check

This check consists of 30 s beam‐on time with an open field $\left(40\times 5\;{\text{cm}}^{2}\right)$ and fixed gantry (0°). It is done with two ionization chambers $\left(0.125\;{\text{cm}}^{3}\right)$ (Standard Imaging, USA) simultaneously. They are put into two different depths (2 cm and 18 cm) of a phantom provided by TomoTherapy (solid water plates, length: 15 cm, width: 55 cm, height: 19 cm) (TomoTherapy Inc., Madison, WI). The value of interest is the dose ratio of the two chambers (value(18 cm)/value(2 cm), similar to the quality index), which should be constant because it is independent from chamber‐based corrections, temperature, and pressure, as well as from machine output variations. We acquired values over a period of about three years.

### B. IMRT check

A second type of measurement was performed by delivering a reference IMRT prostate cancer plan onto an OCTAVIUS phantom (octagon shaped prism, diameter: 32 cm, height: 32 cm) (PTW, Freiburg, Germany). The 2D‐ARRAY seven29 (PTW, Freiburg, Germany) is aligned parallel to the patient table at a depth of 16 cm from the surface of the phantom. The software MatrixScan and VeriSoft (both from PTW) were used to extract the dose of a specific central point for measurements over a time of about 10 months. In contrast to most other machines, the TomoTherapy unit stops irradiation not by reaching a given value of MUs, but after a certain elapsed time. This means, if the (not‐regulated) dose rate is not at the exact level, the performed plan does not exactly deliver the planned dose. Since this measurement depends on absolute dose, we corrected it to the actual number of monitor units. This is very important if the spectral differences are of interest. Without the correction for monitor units, the measured values sometimes show a dose variation of about 2% solely because of the deviation in the dose rate and not because of changes in the spectrum.

### C. Depth‐dose curve check

This check uses a similar setup as the IMRT check. An open field $\left(40\times 5\;{\text{cm}}^{2}\right)$ with fixed gantry (0°) is irradiated onto the OCTAVIUS phantom for 90 s. However, the phantom (with the 2D array inside) is rotated by 45°. Hence, one dimension of the array is aligned along different depths within the phantom. A line of dose points along this dimension can be seen as a depth‐dose curve. We obtained data from the line in the middle of the phantom for a period of approximately eight months. This measurement was also corrected to the actual number of monitor units, as described before.

### D. Simulation

In addition to the measurements, we performed a simple Monte Carlo simulation of the target with Geant4^(^ ^6^ ^)^ (version 9.2.p01, electromagnetic interactions from the penelope package). An electron beam of 6 MeV impinges on the following geometry: the tungsten target with a thickness of 1.00 mm (fresh target) is located in the middle of a slot with a thickness of 3.30 mm filled with water. Downstream of this, there is 5.00 mm of steel and 10.25 mm of aluminum. A quadratic detector (distance from aluminum surface: 80 cm, edge length: 4 cm) measures the energy spectrum of the created photons.

In contrast to this, for the worn out target, we chose a modified target thickness of 0.10 mm instead of 1.00 mm. These values were determined by measuring both the fresh and the worn out targets, as well as the general geometry of the machine. From a theoretical point of view, a thinner target compared to a thicker one can have several effects, causing both a decrease or an increase in the mean X‐ray energy. The reason for a decrease in the mean energy is a lack of photon self‐absorption. In thick targets, self‐absorption vastly reduces low‐energy photons. In the case of a thin target, more low‐energy photons stay in the beam, which therefore results in a lower mean beam energy. Since our results show a decrease in the mean beam energy, we assume that this could be the dominant effect. However, there are two effects that increase the mean energy in a thinner target. First effect: while traversing the material, the electrons lose energy. If the target is thinner, not that much energy is lost and, therefore, the mean energy of the electrons interfering with the material is higher. These higher energy electrons will produce higher energy X‐rays. Second effect: self‐absorption followed by pair production, which is more likely if the target is thicker, decreases the mean energy as well and, therefore, increases the mean X‐ray energy in thinner targets.

## III. RESULTS

### A. Relative dose check

**Figure 1:**
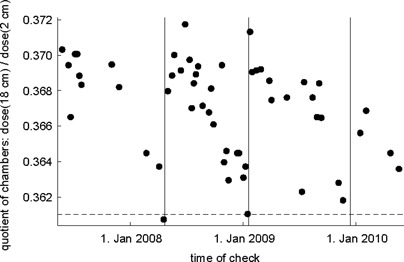
Relative dose check: Dose quotient of two ionization chambers in different depths within the phantom (lower divided by upper chamber). There is a trend of decreasing values within the lifetime of each tungsten target. Times of actual target replacement shown by three vertical lines. Cutoff value for suggested target replacement shown by a horizontal dashed line.

Figure 1 shows the calculated quotient of the relative dose check. Most of the values are higher than 0.366, but less than 0.370. This means a variation of about 1%. There are three time periods, each with a decreasing trend of the quotient. The three periods start with values around 0.370. Eventually every period comes to an abrupt end and the quotient jumps up again to a higher value. In all three cases this coincides with the time of the target replacement (vertical line in the figure). A smaller quotient (decreased dose in the lower compared to the upper chamber) shortly before the target breakdown suggest a softening of the photon beam. The values of the quotient shortly before the jump are around 0.361. Figure 1 shows that very few values are below 0.363 and always occur a few weeks before the target replacement. We suggest to perform the target replacement if the starting ratio (0.370) and the actual value differ by more than 1.5%.

### B. IMRT check

**Figure 2:**
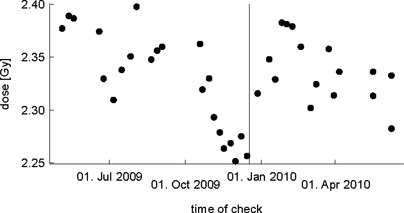
IMRT check: Dose measurement in a depth of 16 cm within a phantom irradiated by a reference IMRT plan. Shortly before the tungsten target is replaced (vertical line), the values drop below the usual range of values.

Figure 2 shows the results from the IMRT check. The values are mostly between 2.31 Gy and 2.39 Gy. From about early November to mid‐December 2009 (after which the target replacement was done), there is a remarkable decrease in the measured dose down to 2.25 Gy. Afterwards the values are again in the usual range. As the measurement is done in a depth of about 16 cm, this confirms the softening of the spectrum. The lowest measured value is about 4% less than the median (2.34 Gy), whereas the highest point is only about 2% higher than the median. We suggest the replacement of the target if the dose decreases by 3% (to 2.27 Gy in our case).

### C. Depth dose curve check

**Figure 3:**
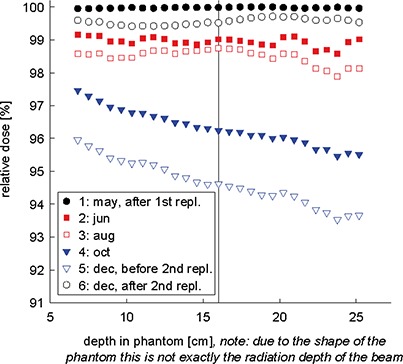
Depth dose curve check: All dose points are normalized to their corresponding values after the first target replacement (measurement number 1). Towards the end of the target lifetime, the doses decrease substantially (numbers 2 to 5). Additionally, the dose points located deeper in the phantom receive the lowest doses with a worn out target (number 4 and 5). The center of the phantom (in a depth of 16 cm) is indicated by a vertical line.

Figure 3 shows the results for the depth dose curve check. For each depth, all dose points are normalized to the value of the reference measurement right after the first target replacement (measurement number 1). The values of every single chamber are decreasing with time (numbers 2 to 5). With the new target installed (number 6), the values are close to the reference values again. For the last measurements before the target replacement (numbers 4 and 5), the deeper the chambers are inside the phantom, the lower the values become (causing a greater discrepancy compared to the reference values). These measurements support a steepening of the depth‐dose curve with time, which again can be explained by a change in the photon spectrum to a lower mean energy. We suggest initiating the replacement of the target when the values at about 13 to 16 cm depth differ by more than 4% from the reference (16 cm is the center of the phantom).

### D. Simulation

TomoTherapy states a mean X‐ray energy of 1.80 MeV for the treatment beam. In our simple simulation, this value was determined to be 1.73 MeV for a fresh target of 1.0 mm thickness and 1.56 MeV for a worn out target of 0.10 mm thickness. Whereas the absolute number for the fresh target does not quite match the one given by the vendor, the decrease of 11% in the mean energy might explain the changes in the depth‐dose curve. As outlined above, the physical reason for a lower mean energy in a thinner target (worn out target) is the lack of photon self‐absorption which otherwise would cause the removal of low‐energy X‐rays.

## IV. DISCUSSION

The results from our measurements indicate a decrease of the mean beam energy towards the end of the lifetime of the tungsten target. This is caused by wear effects that make the target thinner. This effect was also described by Staton et al.^(^ ^5^ ^)^ The performed simulation showed the same effect, but was not designed to give any exact value. Hence, measurements that are sensitive to the photon spectrum are candidates for the prediction of the target breakdown. Our most extensive measurement series is the relative dose check. Therefore, it qualifies as a first predictor for future target replacements. We demonstrated that, for our machine, this is likely to happen when the relative dose quotient for the two different depths (the dose at 18 cm divided by the dose at 2 cm) approaches 0.361. However, depending on who performed the check, we obtained slightly different values, but the results were the same (not shown). The IMRT check confirms the softening of the spectrum since we measured decreasing relative dose values in the middle of the phantom. With this check, we stated a threshold value of 4%. The maximal measured difference to the reference value was about 6%, which is in very good agreement with the results of Yadav et al.^(^ ^7^ ^)^ In the long term, the depth‐dose curve check, which was specifically designed for that purpose, might be the better indicator for predicting a necessary target replacement. However, we only introduced this measurement after the second replacement. Therefore, we have only the data of one replacement.

To predict the time to replace the target, we analyzed three different measurements. The retrospective check of our suggested criteria leads to three different dates in our case, all within three weeks. The target replacement in this special case took place another three weeks later when the target was really broken. This confirms that the suggested measurements and criteria might be good predictors for a necessary target replacement. TomoTherapy engineers perform measurements of the beam (a few times per month) to check the constancy of the output and the energy, as well as the profile. A change in energy will automatically change the beam profile. The data the service engineer obtains from his tests comes from the on‐board detectors collected in one depth. After analyzing the data, the engineer can adjust the energy by changing the injector current. This not only influences the energy of the single electrons, but also the current (and therefore the dose rate). To match the previous dose rate of the machine, the pulse forming network (pfn) can be changed. After the digital adjustment of the values and the resulting required check of the beam quality, treatment can be resumed a few minutes later. In the time before target replacement, a reconditioning of these values is needed (sometimes every week) to get the right dose rate (measured from the on‐board monitor chambers) and the right beam profiles. However, these adjustments of the energy‐related values are limited to a certain range. If these limits are reached, the decrease in energy cannot be compensated anymore. With the measurements done by TomoTherapy engineers, a target wear could be detected, as well. Although these measurements are done, our measurement (performed with different equipment) can show other aspects. With our independent measurements, a degradation in energy can be seen before the target replacement in our machine.

## V. CONCLUSIONS

We present measurements that are designed to predict the tungsten target breakdown for a TomoTherapy Hi·Art machine and suggest a specific predictor for this breakdown. Since the replacement can be handled much easier and faster if the point in time is known beforehand, these measurements, which can be part of the usual QA checks, are useful tools for using a tomotherapy machine more efficiently. The frequency for this QA check in our clinic varies from weekly (or even less) to nearly daily in the last few weeks before target replacement. Our results suggest that the decrease of the target thickness over time leads to a decrease in the mean photon energy (which was also found by other groups). This causes a steeper decline of the depth‐dose curve that can be measured by various depth‐dependent setups.

## ACKNOWLEDGMENTS

Supported by the Bayerisches Staatsministerium für Umwelt und Gesundheit and by the DFG Cluster of Excellence: Munich‐Centre for Advanced Photonics.
